# The Relationship Between Fibroblastic Growth Factor Receptor-1 (FGFR1) Gene Amplification in Triple Negative Breast Carcinomas and Clinicopathological Prognostic Factors

**DOI:** 10.30699/ijp.2019.96713.1952

**Published:** 2019-09-22

**Authors:** Amir Hossein Jafarian, Melika Kooshkiforooshani, Farzane Farzad, Nema Mohamadian Roshan

**Affiliations:** Department of Pathology, School of Medicine, Mashhad University of Medical Sciences, Mashhad, Iran

**Keywords:** Triple-Negative Breast Cancer (TNBC), Fibroblastic Growth Factor Receptor-1 (FGFR1), Immunohistochemistry (IHC), Real Time PCR, Gene Amplification

## Abstract

**Background & Objective:**

In Triple-Negative Breast Cancers (TNBCs), estrogen receptor (ER), progesterone receptor (PR) and HER2/neu genes are not expressed. Fibroblastic Growth Factor Receptor-1 (FGFR1) gene product is a protein that acts as a receptor of thyrosin kinase. It plays a role in the proliferation, differentiation, and migration of malignant cells. The objective was to evaluate the possible relation between FGFR1 over-expression and amplification in TNBCs and other clinicopathological variables.

**Methods:**

In this cross sectional study, purposive sampling was used to collect eighty-four TNBC specimens from mastectomy specimens collected between 2013 and 2017. Tissue microarrays were evaluated for FGFR1 over-expression and amplification respectively by immunohistochemistry (IHC) staining and real time Polymerase Chain Reaction (PCR). The needed clinical and paraclinical information were obtained from patients’ files. To analyze the correlation among prognostic factors, we used a wide range of different statistic methods, namely Chi-square test, independent t-test, Fisher's exact test, and ANOVA.

**Results:**

FGFR1 over-expression was found in 15 of the 84 samples (17.9%). FGFR1 gene amplification was observed in 33.3% (28 of 84) of the samples. We found no association between FGFR1 and clinicopathological parameters, including tumor grade, stage, and patient survival (*P*>0.005).

**Conclusion:**

FGFR1 over-expression and amplification may not be related to clinicopathological parameters, namely age, stage, and grade of the cancer not to mention TNBC survival. Using FGFR1 as a prognostic factor in TNBCs requires further study.

## Introduction

Triple negative breast cancer (TNBC) usually has an aggressive clinical course and is unresponsiveness to anti-HER2 and endocrine therapies (1). Fibroblast growth factor receptor (FGFR) signaling has been noted in several biological processes such as cell proliferation and differentiation, migration (2). Considering FGFR role in cell proliferation, aberrant FGFR signaling has been observed in some malignant conditions (3). For instance, in breast cancer, FGFR aberrations and gene amplifications and consequent FGFR signaling have been associated with worse prognosis and resistance to anti-tumor treatments (2, 3). FGFR1 gene amplification is linked to over expression of FGFR1 (4).

FGFR is a target for the treatment of TNBC, Herceptin-resistant Her2+ breast cancers and tamoxifen-resistant ER+ (positive estrogen receptor) breast cancers. (5). FGFR1 gene amplifications have been studied in other cancers as well (6).

FGFR pathway has been recently studied as a predictive and/or prognostic factor. There is evidence that aberrant FGFR expression is related to a higher likelihood of breast cancer. In addition, expression of FGFR leads to poor prognosis in these patients. There is amplification of FGFR1 and FGFR2 in ER+ breast cancer and TNBC, respectively, and FGFR1 amplification was noted as an independent factor to predict the overall survival in ER + breast cancer (7).

In a study on FGFR1 analyses regarding gene copy number and its relationship with clinicopathological parameters among ER-positive/HER2-negative primary breast cancer, a high level of FGFR1 expression was detected in about one-fourth of the subjects. Furthermore, a poor relapse-free survival rate was found to be associated with high FGFR1 expression. 

FGFR1 gain/amplification was found in 14% of the patients (8).

FGFR1 amplification is known to have meaningful association with worse prognosis among breast cancer patients, in particular those with ER-positive cancers. One study reported that FGFR1 amplification confers resistance to therapy. FGFR1 signaling promoted cyclin D1 expression and suppressed progesterone receptor expression. About one-fourth of breast cancers potentially expressed FGFR1 amplification. In such examples, FGFR1 may represent an alternative signal to resist endocrine therapies (9, 10). Increased FGFR1 expression has also been shown to have relationship with lobular breast cancer (11).

TNBC is an aggressive malignancy which is diagnosed usually in younger women and suitable targeted therapy is not yet introduced for TNBC. In this article we try to review the clinicopathological features of TNBC and any possible prognostic relationship or therapeutic potential of FGFR1 as an emerging targeted therapy. Inhibition of FGFR signaling is being studied extensively and targeting FGFR could be a potential treatment for TNBC. 

## Materials and Methods

**Fig. 1:**
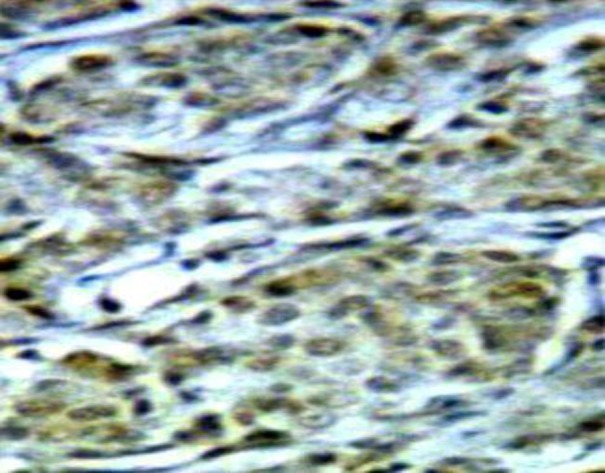
Severe colorfulness of FGFR1 expression in IHC

**Fig. 2:**
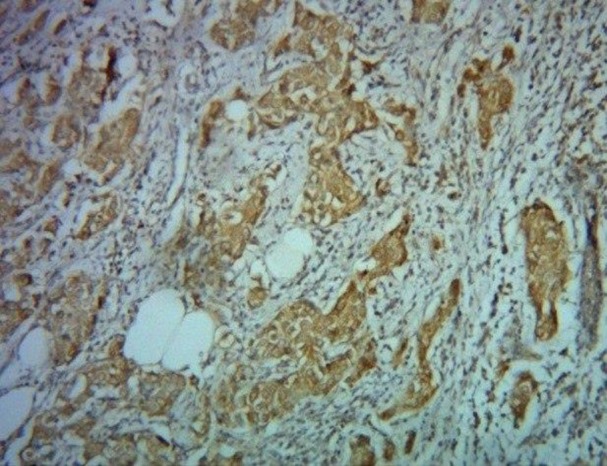
Weak colorfulness of FGFR1 expression in IHC

In this cross-sectional study, female patients diagnosed with TNBC who underwent mastectomy at our medical center from 2012 to 2017 comprised the study population.‌ Inclusion criteria comprised living patients 4 weeks after surgery who have completed information of the required clinicopathological factors and given an efficient sample for IHC (immunohistochemistry) and real time PCR (polymerase chain reaction). A data collection checklist was designed and the required variable including age, histopathologic diagnosis, tumor grade, tumor stage and months patient survived after being diagnosed were documented. Eighty-four patients were included. The interval between diagnosis and death or phone call (if they were alive) was considered the survival time. Subsequently, paraffin blocks with sufficient tumoral tissue were taken and two pathologists checked the tumors’ grades. For IHC, 3×4 micron slides were provided and stained by rabbit polyclonal antibody [anti FGFR1 (phospho Y654) antibody cat (No: ab59194, ABCAM, Cambridge, UK). Like similar studies, we have used lung adenocarcinoma tissue as a negative control and lung SCC as a positive control. In terms of the percentage of colorfulness, cells were divided into 5 subsets: under 1%, 1-25%, 25-50%, 50-75%, 75-100% which received scores ranging from 0 to 4, respectively, and the degree of colorfulness was categorized as negative, weak, moderate, severe with scores from 0 to 3. For each sample, the degree of colorfulness was multiplied by the percentage of colorfulness, and the received number was called the FGFR1 expression score for that sample. Therefore, samples were scored from 0 to 12 in terms of FGFR1 expression. Like other studies, scores from 2-12 were considered as positive samples (Figures 1 and 2).

Below is a brief explanation of IHC steps, similar to other studies and based on a standard process (12??

We blocked endogenous peroxidase action by using hydrogen peroxide 5% for 5 minutes at room temperature, then washing for 5 minutes in TBS. In the proprietary antibody attachment step, we put FGFR1 antibody 1/1000 for 90 minutes in the moist dish, and then washed with TBS for 5 minutes. The following step is secondary antibody attachment, in which samples were put for 30 minutes in anti-rat envision horseradish peroxidase antibody, and subsequently washes for 5 minutes with TBS. To see antigen expression, we put samples in Diaminobenzidine which acts as a substrate for peroxidase enzyme, in room temperature for 10 minutes and then we washed samples in TBS for 5 minutes. Finally, after putting samples in non-alcoholic hematoxylin for a few seconds followed by washing, we observed coloring under the microscope. Alcohol (70% up to absolute) and xylenol were respectively used to dehydrate and clarify the samples. At the end, we dried the plate and stuck lamel on it to preserve it.

The section below provides a brief explanation on real time PCR steps as conducted in another similar study (13):

1. DNA extraction from paraffin block of tissue fixed in formalin.

2. Designing primers (direct and reverse) for FGFR1 gene and the control gene, GAPDH.

3. Providing master mix (Tag DNA Polymerase) after primer attachment to single strand DNA proliferates DNA.

4. Providing SYBR Green I, which exclusively bonds double-stranded DNA, fluorescent light is then generated and identified by device. 

5. Real-time PCR proliferation of FGFR1 gene by ABI thermal cycler; 1. Denaturation 2. Annealing 3. Polymerization 4. Repetition of these steps up to 40 cycles so that a significant amount of DNA is synthesized.

Real-time PCR steps include:

1. Linear ground phase: until 16^th^ cycle, the amount of double-stranded DNA is low. Thus, there is no fluorescent light.

2. Early exponential phase: from the 17^th^ cycle, the number of DNA copies is increased so that the device can identify the fluorescent light from the attached SYBR Green I (Threshold cycle).

3. Log-linear phase: by continuing Real-time PCR cycles, the number of double-stranded DNA is progressively increased, as well as the intensity of fluorescent light.

4. Plateau phase: there is no production of DNA. The emitted fluorescent light will stabilize.

5. Check positive amplification, we use Relative Quantitative Real-time PCR in this study. Therefore, like similar studies, positive samples were considered to be those with fold change >2. (The ratio of double-stranded DNA copies of FGFR1 gene to GAPDH gene copies).

For evaluation of fold changes in each sample deltaCt was calculated for FGFR1 and GAPDH and subsequently;

1. Patients group 

FGFR1 gene ∆Ct = FGFR1 gene Ct – GAPDH gene Ct

2. Control group 

FGFR1 gene ∆Ct = FGFR1 gene Ct – GAPHD gene Ct

Ct∆∆ = 1-2

In the Ct∆∆ formula, the number 2 is equal to the efficiency of PCR being at the best and the most ideal situation of the reaction (100%). In order to determine PCR efficiency, a series of different dilutions of DNA copies of FGFR1 and GAPDH genes was provided. After the reactions were conducted and the standard curve drawn, the line slope is seen. Efficiency was = [10(-1/slope)]-1; thus, the efficiency for FGFR1 gene and GAPDH gene was 97% and 96% respectively (Table 1).

**Table 1:** The sequence of direct and reverse primers of FGFR1 and GAPDH genes

Reverse primer (5’…3’)	Direct primer (5’…3’)	Gene
TGTCACCAGCCTTCATGACC	ATGGTTGCAGGTGATGTGGT	FGFR1
TTCAGCTCAGGGATGACCTT	CCACCCAGAAGACTGTGGAT	GAPDH

Fusion curve: concerning the usage of SYBR Green I in our study, a fusion curve was drawn for both FGFR1 and GAPDH genes. As seen, this curve has only two peaks, one for FGFR1 and the other for GAPHD. So there is no nonspecific product in our test.

**Statistics**

For analysis of the correlation among prognostic factors, the Chi-Square test, Student T-test, Fisher's exact test, ANOVA and Cohen's kappa coefficient were used. All analyses were performed using SPSS 16.0 (SPSS Inc., Chicago, IL., USA). The final sample size was estimated to be 84 with a 30% prevalence of amplification in similar studies, a 95% safety factor and 30% relative precision.

**Ethics**

The study protocol was verified by the Research Council Ethics Committee of Mashhad University of Medical Sciences. No informed consent was required and mastectomy was indicated for the patients. The study was in conformity with the Declaration of Helsinki. 

## Results

The mean (±SD) age of the patients was 50.93 (±12.17) years (range, 26 to 88 years). Age had a normal distribution (*P*=0.420). 

The mean follow-up period was 17.5 months (range, 3 to 38 months). The frequency of clinicopathological variables is shown in Table 3. The frequency of the FGFR1 expression score was as follows: 0-1 (69 samples, 82.1%), 2 (one sample, 1.2%), 4 (four samples, 4.8%), 6 (four samples, 4.8%), 8 (four samples, 4.8%), and 9 (two samples, 2.4%). 

There was no significant correlation between FGFR1 gene amplification and tumor stage (*P*=0.116) as well as tumor grade (*P*=0.549) (Table 4). Likewise, no statistically significant difference was seen regarding FGFR gene over-expression based on tumor grade (*P*=0.640) and clinical stage (*P*=0.116) of TNBCs (Table 3). 

**Fig. 3:**
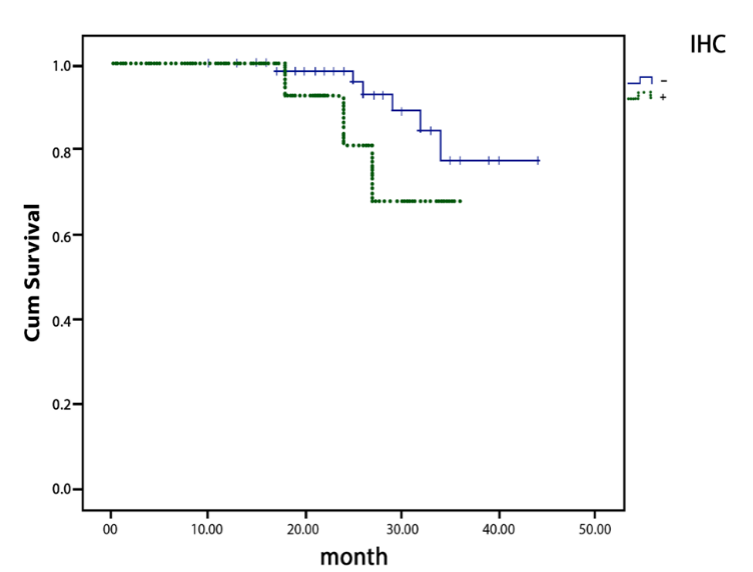
Overall survival curves according to the result of FGFR1 gene overexpression

**Fig. 4:**
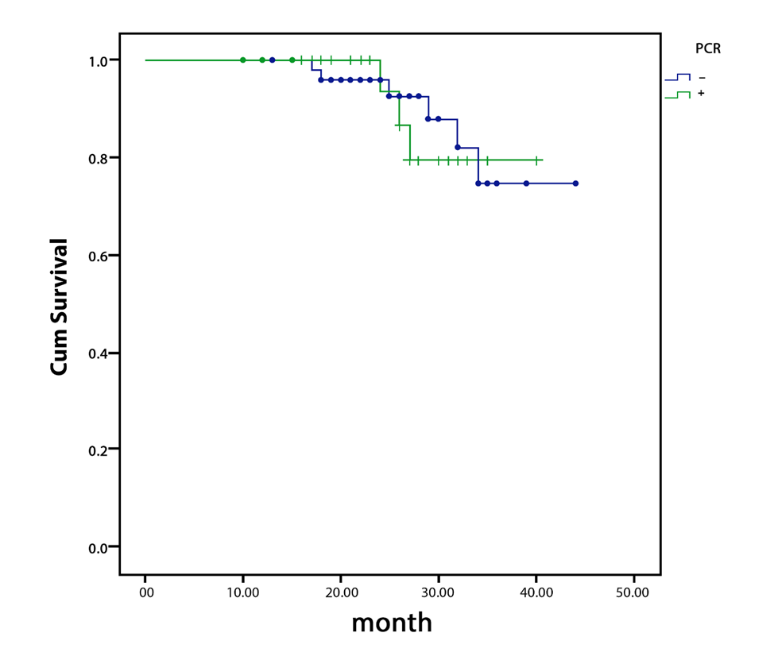
Overall survival curves according to the result of FGFR1 gene amplification

According to Kaplan-Meier analysis, there was no significant correlation between FGFR1 gene amplification and survival rate (*P*=0.885) (Figure 3). Similarly, FGFR1 overexpression did not have significant correlation with the survival of the patients (*P*=0.157) (Figure 4).

**Table 2:** Clinicopathological characteristics of 84 female patients with Triple Negative Breast Carcinoma (TNBC)

		No. (%)
Tumor grade	1	7 (8.3%)
	2	35 (41.7%)
	3	42 (50%)
Stage	1	2 (2.4%)
	2	36 (42.9%)
	3	35 (41.7%)
	4	11 (13.1%)
Survival	Alive	75 (89.28%)
	Died	9 (10.71%)
IHC	Positive	15 (17.9%)
	Negative	69 (82.1%)
PCR	Positive	28 (33.3%)
	Negative	56 (66.7%)

**Table 3:** Comparison of FGFR1 over-expression according to tumor grade and stage of 84 patients with TNBCs

		FGFR1 gene amplification		P-value
		Positive	Negative	
Tumor grade	1	2 (2.3%)	5 (6%)	0.640
	2	5 (5.95%)	30 (35.71%)	
	3	8 (9.52%)	34 (40.47%)	
Stage	1	0	2 (2.3%)	0.116
	2	8 (9.52%)	28 (33.33%)	
	3	5 (5.95%)	30 (35.71%)	
	4	2 (2.3%)	9 (10.71%)	

**Table 4:** Comparison of fibroblastic growth factor receptor-1 (FGFR1) gene amplification according to tumor grade and stage of 84 patients with TNBCs

		FGFR1 gene amplification		P-value
		Positive	Negative	
Tumor grade	1	2 (2.3%)	5 (6%)	0.549
	2	14 (16.9%)	20 (24.1%)	
	3	12 (14.5%)	30 (36.1%)	
Stage	1	0	2 (3.3%)	0.116
	2	17 (19.54%)	19 (22.6%)	
	3	9 (10.71%)	26 (30.95%)	
	4	29 (34.52%)	9 (10.71%)	

## Discussion

Here, we decided to determine the prognostic value of FGFR1 over-expression and amplification in TNBCs. According to the obtained findings, FGFR1 over-expression and amplification did not have significant associations with the studied clinic-pathological parameters. As FGFR1 as well as FGFR2 are expressed in about 10% of breast cancers, these have gained attention for several purposes, including targeting newer treatments against these factors and using these as determinants of prognosis and overall survival of the patients. As TNBCs have generally worse prognosis compared to other types of breast cancers, this type of tumor needs further investigation for better clarification regarding various genes, including FGFR. 

In the last two decades, growing evidence has been published in the literature regarding our knowledge on the molecular heterogeneity of breast cancer. Still, TNBC remains a malignant condition with poor prognosis. Fibroblast Growth Factor (FGF) signaling deregulation has been noted in breast cancer, and finding ways to block this deregulation has been advocated as potential therapeutic methods (14). We therefore decided here to determine FGFR1 expression in TNBC. 

In a Nedeljkovic* et al. *study, FGFR1 as well as c-MYC gene copy number alterations were studied in samples obtained from 78 patients with TNBC. For this purpose, they applied TaqMan-based quantitative real time PCR and about 43% of the samples had an increased FGFR1 copy number. Similar to our study, no meaningful correlation between the FGFR1 copy number gain and clinicopathological variables was observed. They noted that FGFR1 gene copy number has a low prognostic importance in TNBC (15).

In a separate study to explore the role of FGFR expression in TNBC, in contrast to other reports, FGFR1 was in fact a prognostic value for overall survival in TNBCs (16).

There is controversy in the literature regarding the prognostic value of FGFR1 in TNBCs. For example, Lee* et al. *evaluated 148 primary TNBCs for FGFR1, FGFR2, and FGF2 protein expression by FISH method. They showed that FGFR1, FGFR2, and FGF2 expression were found respectively in 16.2%, 12.8%, and 12.8 % of TNBCs. FGFR1 gene amplification was observed in 4.1% of the cases. FGFR2 expression was found to be associated with basal-like TNBC. Compatible with our results, the mentioned study did not find any significant association between FGFR1 expression and clinicopathological factors. Only a subset of patients whose tumors expressed FGFR2 expression was shown to have a lower histological grade. None of the examined factors were shown to have implications for the survival of the patients (14). 

A different approach to investigate the role of FGFR1 in TNBCs was a study that examined the sensitivity of a panel of 31 breast cancer cell lines to the selective FGFR inhibitor PD173074. In comparison to comparator cell lines, cell lines of TNBC were more sensitive to PD173074. This observation reflected significantly reduced growth. The majority of TNBC cell lines had moderate sensitivity to FGFR inhibition in two-dimensional growth, but was highly sensitive in anchorage independent conditions (17).

In another study including 148 primary TNBCs, none of FGFR1, FGFR2, and FGF2 expressions had implications for outcomes (18).

## Conclusion

Triple-negative breast cancers (TNBCs) are clinically aggressive tumors with limited treatment options. On the other hand, FGFR1 is relatively frequently amplified and over-expressed in breast and lung cancer and is currently widely accepted to have a carcinogenic effect. However, it seems there is no correlation between FGFR1 over-expression and amplification and TNBC’s overall survival, tumor grade and tumor stage.
